# Perceptions of Medical Undergraduate Students on Curricular Changes in Anatomy: An Embedded Design Mixed Method Study

**DOI:** 10.30476/JAMP.2021.92149.1472

**Published:** 2022-01

**Authors:** APARNA MURALEEDHARAN, SARANYA RAGAVAN, NUTAN NALINI BAGE, REMA DEVI

**Affiliations:** 1 Department of Anatomy, Pondicherry Institute of Medical Sciences, Puducherry-605014, India; 2 Department of Anatomy, Jawaharlal Institute of Post Graduate Medical Education and Research (JIPMER), Karaikal, Pondicherry – 609602, India

**Keywords:** Problem–based learning, Anatomy, Curriculum, Medical education, Learning, Teaching

## Abstract

**Introduction:**

Implementation of competency-based medical education (CBME) offers challenges to the faculty and students. As a part of the new curriculum by the National Medical Council of India, we introduced certain teaching and assessment modifications in anatomy. There are few studies on the actual implementation of CBME. The current study aimed to assess the perceived usefulness of the teaching, learning, and assessment methodology (TLAM) based on written feedback from students.

**Methods:**

All the 147 first MBBS students of the batch who had undergone the new teaching learning methodology answered the questionnaire on usefulness of the various TLAMs on a 3-point Likert scale which was taken as quantitative data. They were also asked to pen down their opinions and suggestions about the TLAM which was thematically analysed and considered as qualitative data. Face and content validity was assessed prior to administration of the questionnaire. Descriptive statistics were used for quantitative variables. Qualitative data were analysed by thematic analysis.

**Results:**

100% of the students found vertical integration and small group teaching as useful. Seminars and quizzes were reported as useful to extremely useful by 75.5% of the students. Self-directed learning and near-peer teaching was also appreciated well by 78% of students. In Embryology and Neuroanatomy, these active learning methods were not found to be useful as these topics were difficult to understand. Overall, the new TLAM introduced as per the new medical curriculum was found useful for the majority of students.

**Conclusion:**

This study provides valuable insights on the teaching, learning, and assessment methods as formulated by the competency-based medical curriculum. Though active learning methods are the integral principles of andragogy, the concepts which are difficult to understand need to be taught using the traditional teaching methodology.

## Introduction

The undergraduate curriculum in India has seen a drastic shift in the last few academic years. The concept of competency-based medical education (CBME) was first put forth by Mc Ghahie in 1978 ( 1 ). The new CBME by National Medical Council of India aims to be more learner-centric, patient-centric, gender-sensitive, outcome-oriented, and environment- appropriate ( 2 ). It is an outcome-driven undergraduate curriculum that has elements which encourage life-long learning. It is competency-based and not time-based like the traditional curriculum. The focus has shifted from traditional didactic lectures to self-directed learning or a flipped classroom approach. The crux of the CBME involves active student participation and engagement and team-based learning (TBL). Early clinical exposure (ECE), addition of elective subjects, acquisition of certain skills, development of the right attitude, ethics, communication (AETCOM) skills, self-directed learning (SDL), and addition of co-curricular activities are all components of CBME. Its major emphasis is on alignment and integration of all possible subjects both horizontally and vertically, so that the students will be able to realise the importance of a topic in clinical practice. This contrasts with the traditional medical curriculum, where the stress is on knowledge acquisition and all subjects in a particular year are introduced simultaneously – preclinical subjects in the first professional course, paraclinical subjects alongside clinics in the second professional course, and purely clinical subjects in the subsequent years. This method has been replaced by the new CBME to provide personalised holistic care to all patients ( 3 ).

Implementation of CBME offers challenges to the faculty and students as there is major restructuring of the traditional pedagogic approaches ( 4 ). In anatomy, the method has always been team-based learning (TBL) and learning by doing ( 5 ). Clinical anatomy where normal anatomy is applied to daily life and clinical practice is always introduced right in the first professional medical course. Anatomy is the first introduction to true or real medicine ( 6 ). Even in traditional method, lectures in anatomy are always followed by corresponding laboratory training in the dissection room or in the histology laboratory. Thus, the students who acquire the skills of *Know* and *Know How* during the lecture easily acquire skills to *Show*, *Show How* and *Do* during the practical session ( 7 ). The curricular changes are easier to implement in anatomy as it is the starting point of professional medical education when a senior secondary school student shifts to college education. A good knowledge of anatomy is necessary for the understanding and practice of clinical subjects ( 8 ). Hence, it is necessary to modify the teaching and evaluation methods in all subjects, especially anatomy.

As a part of the new CBME curriculum by the Medical Council of India, we introduced necessary teaching and assessment modifications in anatomy. There are several reports comparing traditional method and CBME. However, there are only few studies on the actual implementation of CBME. The current study aimed to assess the perceived usefulness of the teaching, learning, and assessment methodology (TLAM) based on written feedback from students. The methods were also continued during the online anatomy teaching in the wake of COVID-19. Since CBME is only taking its baby steps in several countries, we expect that this study would give valuable ideas for future curricular modifications. We hope that this study would lay a roadmap to plan and prepare for curricular reforms in future.

## Methods

**Figure 1:**
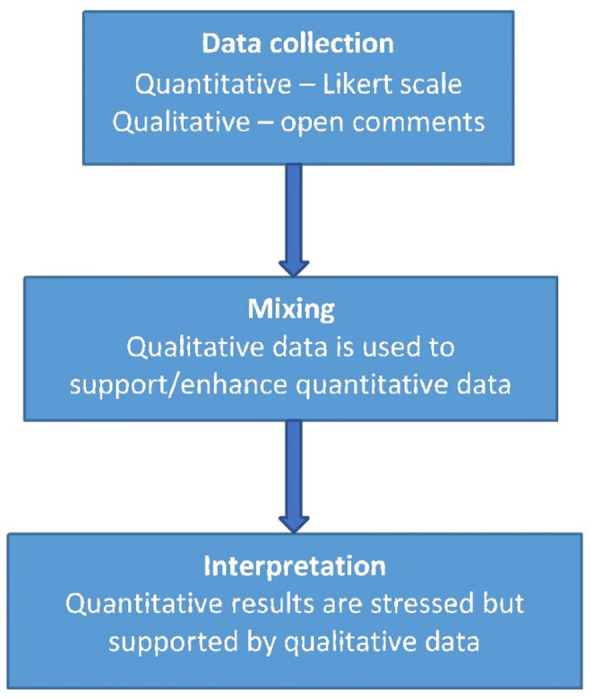
Embedded design mixed method study design

The study was conducted after the exams to avoid information bias and infringement of autonomy. The aim was to explore the perceptions of students about various TLAMs. All the 147 first MBBS students of the batch who underwent the new teaching, learning methodology and appeared for the first professional MBBS examination answered a pre- designed pre- validated questionnaire where items were aligned with the research question. Kirkpatrick level I framework was used for evaluation. A self-administered feedback questionnaire was used for the study. The questionnaire was tested for face and content validity. Face validity was assessed by a subject expert. Content validity was assessed by five experts (3 from anatomy and 2 in medical education). The experts assessed the content for 1) relevance of each question, 2) clarity, 3) necessity/essentiality, and 4) suggestions to improve/add/remove questions. Based on the recommendations by the content experts, the questionnaire was revised three times and the 3rd version was taken as the final version for the study. There were 45 items in the questionnaire which contained questions regarding the usefulness of various TLAMs. Of the 45 items, 34 were marked as very relevant by the content experts. The study was an evaluation of the perception of all new TLAM performed during the academic year, and the study was conducted after the completion of university exams as per the mandate by the institute Ethics Committee. Thus, the same participant could answer the question only once. Hence, test-retest reliability was not assessed. The questions were on self-reported usefulness of various TLAMs on a 3-point Likert scale (1 - Not useful; 2 – Useful; 3 – Extremely useful); the quantitative data are expressed as proportion. Qualitative data were collected by asking the students to write down their opinions and suggestions about the TLAMs that were used. They were also asked to write down suggestions to modify and/or improve them. Only open-ended questions were asked regarding various TLAMs. Qualitative data were analysed after thematic analysis. The recurring themes were assessed. The themes were generated by the first author and validated by the second one. In the analysis stage, the qualitative data were used to substantiate the quantitative data; hence, it is an Embedded design mixed method study (Figure 1).

The TLAMs that were evaluated are:

1. Lecture classes - Conventional teaching, teaching using clinical case vignettes, videos, animations.
2. Integration – Horizontal integration with Departments of Physiology and Biochemistry, Vertical integration on clinically relevant anatomy topics by clinicians from relevant clinical departments (Surgery, Orthopedics, Otorhinolaryngiology).
3. Early clinical exposure (ECE).
4. Dissection – Self - dissection, prosecution, team-based learning (peer group discussion, peer teaching).
5. Active learning methods - Daily short seminars on topics covered on the previous day, region-wise seminars on a few large topics covered in that particular region and frequent weekly or fortnightly quizzes, small group discussion (SGD), team based learning (TBL) near-peer teaching, self-directed learning (SDL)
6. Preparation and priming methods - PowerPoint projection, short videos, clinical vignettes prior to dissection, histology practicals, small group discussion, self-directed learning.
7. Embryology and radiology - Embryology classes were taken using PowerPoint sessions using case vignettes and animated videos. The students would then be divided into small groups for peer group discussion and SDL.
8. Assessment - Formative assessments (FA) were conducted once weekly as objective or short answer tests after which a facilitator would provide feedback to the individual students at the end of the session. Summative assessments (SA) were conducted as traditional Theory and Practical examinations in the prescribed university pattern.
9. Attitude ethics and communication (AETCOM) - Apart from the orientation programme conducted by the institute, the anatomy faculty conducted a cadaver disrobing ceremony in the beginning of the academic year and robing ceremony at the end of the year to create awareness of the body donation programs and develop a feeling of the human donor as their first patient. This session included videos, audios and feedback by faculty and students about their perceptions on human donor and dissection. Sessions on professionalism, ethics and humanities in medicine were also conducted as part of the AETCOM module to teach them the importance of the medical career, and history of Modern Medicine and dissection.
10. Electronic media - The various electronic media used in learning include e-learning platforms like Moodle and WhatsApp groups where quizzes are posted, and the students can clear their doubts. Various methods of SDL, FA, SA, Integration, ECE, small group teaching were conducted using online platforms like Google Meet, even during online classes.

### Ethical Consideration

The study was approved by the Institute research committee and Institute ethics committee (CDSCO- Reg No. ECR/400/Inst/Py/2013).

## Results

**Figure 2:**
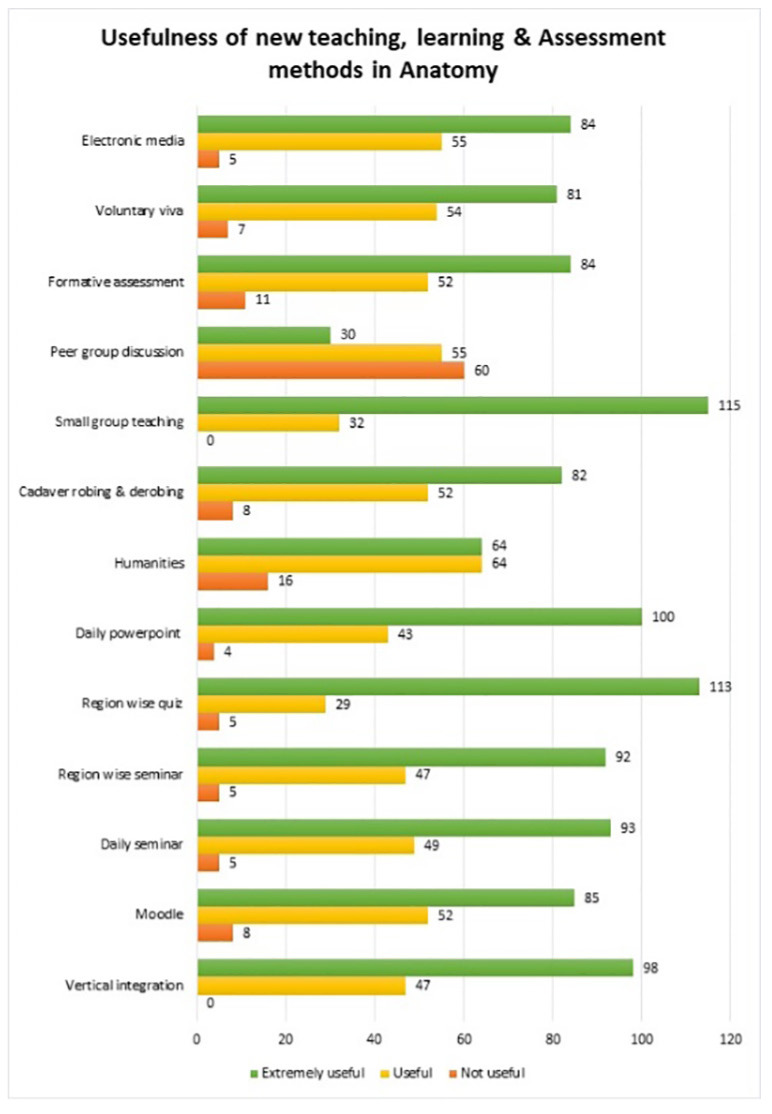
Usefulness of various Teaching Learning and Assessment methods as rated on Likert Scale

Of the 147 first MBBS students who participated in this study, 92 (62.58%) were girls and 55 (37.41%) were boys. As it was program evaluation, the response rate was 100%. The usefulness of various TLAMs is given in Figure 2. The suggestions and comments by students on various TLAM are given in Table 1. The thematic analysis of students’ feedback regarding usefulness of various TLAM is given in Table 2. The striking findings of our study is as follows:

**Table 1:** General Comments/suggestions by students on various Teaching, Learning, and Assessment methods

Sl.no.	Students’ comments
1	We need more clinical pictures and case videos in lecture.
2	We need more clinical case-based teaching.
3	We need more lectures integrating with clinical subjects especially when the patients are brought to the class, or we are taken to the wards. It is very much interesting and extremely interactive. We never forget what we see.
4	We need more seminars, quizzes, and group discussions. These were useful for last minute preparation for university examinations.
5	We need short weekly test / short spotters test after each topic apart from the routine monthly internal assessments.
6	We need videos at the start of every dissection class which gives us an overview of the region to be dissected.
7	Embryology is difficult to understand, especially general embryology. Apart from routine lecture class the teachers should first give an orientation during small group teaching followed by our peer group discussion.
8	We need more clinical case scenarios-based essays in internal assessment.
9	We need more formative assessments. Such short questions were useful for answering short notes during university examination.

**Table 2:** Major recurring themes in qualitative analysis

Methodology	Students’ comments
LECTURE CLASS	
81% - clinical case vignette-based lecture classes – extremely useful	“Beginning a class with a case scenario is thrilling and I feel that I am solving a case by the end of the class. It’s an excellent method of set induction”.
50% - Blackboard teaching is useful	“When teachers write or draw on blackboard, I am able to better understand and draw the diagrams easily than when shown in PowerPoint”.
INTEGRATION	
67% - vertical integration – extremely useful	“When a clinician comes to speak of an applied anatomy, I feel that I am more interested, especially when they talk of cases they have seen and treated in our institute. We were so interested in the sessions where they brought patients to the classroom and some of us got the opportunity to talk and examine them. It is interesting and interactive. We never forget what we see.”
DISSECTION	
69% - Self dissection is extremely useful	“I prefer doing dissection myself as it gives me a better understanding of the orientation, course and relations of structures than when a teacher does dissection or teaches on a prosected specimen. But it is extremely useful if the dissection is preceded by a small introduction by the faculty or a dissection video prior to the self-dissection”.
SELF-DIRECTED LEARNING	
75.5% - SDL and group discussion in dissection hall is extremely useful	“After lecture and dissection of a particular topic, SDL and group discussion is very useful for learning most of the topics in gross anatomy, except a few difficult ones”.
60% - SDL is not useful for embryology and radiology	“Even after attending lecture classes, I have found that Embryology is very difficult to understand by SDL as well as peer group discussion”.
93.3% - daily seminar as useful to extremely useful	“I used to prepare sincerely for the daily seminar topics which are given to us well ahead of time in the monthly schedule, and I must say that I have never revised them for my final exams as I was thorough with the topics”.

***Lecture class:*** 81% (n=119) of students found lecture classes using clinical vignettes as extremely useful. Majority of the students wanted lecture classes using PowerPoint incorporating videos and pictures of clinical cases. 50% (n=74) of students wanted blackboard teaching along with the PowerPoint as it helped them to draw diagrams easily. 

***Integration:*** 67% (n=98) found that integration with other departments is useful. They felt that vertical integration with clinical departments is extremely useful. Integration sessions in which the patient was brought to the class was more useful as they could see the clinical presentation in real compared to theoretical teaching. Horizontal integration was done for most of the regions like Limbs (with musculoskeletal physiology), Thorax (with respiratory and cardiovascular physiology), Abdomen, Pelvis & Perineum (with gastrointestinal and genitourinary physiology), Neuroanatomy (with neurophysiology). 

***Dissection:*** 69% (n=101) of students found self-dissection as extremely useful. Majority of these students wanted a faculty to give them orientation of the region to be dissected or demonstrate on a prosected specimen before the dissection. 

***Active learning methods, SDL:*** Self-directed learning (SDL) and group discussion were reported as extremely useful by 75.5% (n=111) of students for topics in Gross Anatomy. 60% (n=88) of the students reported that SDL and near-peer teaching was not found to be useful for embryology and neuroanatomy as the concepts in these topics were difficult to understand. 77% (n=113) of students found active learning methods like daily seminars and frequent quizzes as extremely useful. Many of the students commented that the topics that were given for daily seminars were never revised after the seminar as it was thorough for them.

***Assessment pattern:*** 87% (n=128) of students found the pattern of formative and summative assessments as extremely useful and comparable with that of their university examinations. The assessment pattern was satisfactory as they found that it correlated well with the University exam pattern.

***Online learning:*** 57% (n=84) of students found electronic media and e-Learning platforms as extremely useful. Majority of students used these platforms to submit assignments and clarify doubts with each other and with the concerned faculty. 

## Discussion

Competency-based medical education (CBME) has been in practice for several years in many developed nations, but it is taking its baby steps in some other countries like India only recently. Since there is extremely limited information on the actual implementation of CBME, this study was done to evaluate the usefulness of the new CBME-based TLAM to lay a roadmap for future curricular reforms. Clinical case-based classes, self-directed learning methods, peer group discussion, small group teaching, integration, early clinical exposure, and active learning are the highlights of the new CBME, and these were well received and found to be extremely useful by most of the students. This has been shown in some previous studies as well ( 9 ).

Proper theoretical knowledge and strong base in anatomy is essential before clinical practice. The conventional pedagogical lecture classes were replaced to a large amount by active learning methods ( 10 ). Small group teaching was found to be extremely useful for the students when compared with the traditional lectures. This has been reported in previous studies as well. Hommes et al. have reported that better group learning processes can be achieved by grouping large classes into small groups ( 11 ). This raises the importance of small group teaching and better teacher-student and student-student interaction, which is possible only if the group is small and the teacher/student ratio is small. 

The active learning methods that were introduced were small group discussion, near-peer teaching, seminars, quizzes, flipped classroom, and SDL. These were well received by most of the students. Literature shows that near peer teaching is a well-established active learning method in medical education. This has been found to be highly effective in previous studies because when their peers teach, slow learners get an opportunity to clear any doubts which they may hesitate to do with a faculty ( 12 ). Evans et al. found that near-peer teaching was an effective method in teaching anatomy. The student teachers also have several advantages. Feedback from other students and teachers, awareness of one’s own positive and negative qualities, improvement in communication techniques, ability to recognize problem learners and rectify them with the assistance of a faculty are some of them ( 13 ). Embryology was reported as the most difficult to understand section of anatomy by most of the students. In our study, we found that small group discussion and near-peer teaching were not useful and not effective in embryology, neuroanatomy, radiological anatomy, and some difficult concepts in gross anatomy as they did not get conceptual clarity. This has been reported for subjects like neuroanatomy in the past ( 14 ). SDL and flipped classroom are integral elements of andragogy ( 11 , 15 ). Different active learning methods may be useful in different subjects ( 16 , 17 ). In our study, the students reported that the small seminars and weekly assessments were extremely helpful for them during their preparation for the final examination. Importance of such active learning methods like spotters, quizzes, and seminar has already been reported ( 18 , 19 ). 

Integration was found to be an effective way to retain and correlate information ( 20 ). When vertical integration was done by bringing real patients to lecture class, the students found it extremely useful. They suggest that the number of Vertical Integration sessions needs to be increased and wanted more of clinical case scenarios, videos, and pictures of clinical conditions in traditional classes as well. This gave them a better interest in listening to the classes as it would have given them better set induction ( 21 ). Horizontal alignment of topics with physiology helped the students to correlate the structure and function of a particular organ better. 

The most salient aspect of anatomy learning is the cadaveric dissection ( 22 ). In our study, majority of the students found that dissecting by self was extremely useful in remembering the anatomy of the structures. Those students who found dissection as not useful were mostly of the opinion that they could not correlate the two-dimensional pictures in atlases or textbooks with the three-dimensional orientation seen in the body and found dissection as difficult. Majority of students reported that dissection had to be combined with small group discussion and team-based learning for better understanding of the concepts and better remembering of information. This has also been reported in previous literature ( 6 , 23 ). Studies have shown that such problems can be curtailed by adequate priming and preparation using lectures, videos, and pre-dissection faculty guidance ( 6 , 24 ). Most students found daily priming in dissection hall in the form of MS PowerPoint projection, videos and displaying of objectives for the day apart from the usual teaching as useful. Majority of students also reported that lecture classes just preceding the dissection session were useful.

Dissection helps to attain the learning objectives and core competencies of an Indian Medical Graduate (IMG) ( 25 ) by encouraging active student engagement, small group discussion and communication skills, and promotes principles of professionalism like altruism, accountability, respect and integrity, teamworkand leadership ( 26 ). The only negative feedbacks received were physical and psychological disturbances. Integration of dissection with new TLAM and technology has curtailed most of the problems ( 22 , 27 ). Dissection also has an emotional influence on the dissector as it provides deep insight into the meaning of death and human body, value of human life, idealistic view of future doctors to deal with real life patients as they call the human donor as their “first patient”.

Due to the advent of the COVID-19 pandemic, the face-to-face classes have reduced, and medical education has shifted to an online mode. The most important facet that is affected is the cadaveric dissection. Dissection videos and virtual dissection help students to get an understanding of the normal anatomy ( 28 ), but this cannot replace the real time dissection hall experience.

The formative and summative assessment methods were also found to be extremely useful for the students. They found that this made them more familiar with various question patterns, assisted them in better time management and better presentation style in exams. Overall, the comments and feedback given by students had more emphasis on the effect of various TLAMs on their performance in the final examination rather than in acquiring and retaining of knowledge. The students considered each TLAM as useful when it positively influenced their exam performance and not useful when it negatively affected exam performance. This trend has been reported in previous studies too ( 29 ). Therefore, the actual effectiveness of all the TLAMs and thus the curricular reforms can be assessed completely only in the long run ( 30 ).

### Strengths and Limitations

The entire batch of first professional year students underwent the modified teaching methodology. Hence, the results may be generalizable to other colleges that have adopted the new curriculum. Therefore, the current study has put forth a stepping stone to several other institutes in new curricular modifications. However, the current study had some limitations. As the study had a cross-sectional design, the participants were not followed up. The perception could have been compared with their final scores, which was not done. The qualitative model used is an embedded design model which is less robust than other qualitative methods. 

## Conclusions

This study gives valuable insights on the teaching learning and assessment methods as formulated by the new competency-based medical curriculum. Small group teaching was found to be extremely useful, particularly for difficult areas in anatomy. Self-directed learning, group discussion, and peer learning in difficult topics was reported as not useful. Hence, active learning methods should be planned considering the topic. The best method of teaching is multi-model teaching as no one method is ideal or complete. Integration is necessary throughout the medical curriculum. Most of the comments about the teaching learning and assessment methods were based on the usefulness in exams to score marks rather than actual assimilation of knowledge and information. Constant change and innovation are required in teaching and assessment to improve understanding of anatomy. We need to devise strategies to increase motivation, awareness of relevance of the subject, and a feeling of competence in the subject which will eventually enhance the learning process to meet the requirements of CBME. Curricular reforms should be a continuous process considering the requirements of all the stakeholders. 

## Acknowledgement

We thank the first year MBBS students for their cooperation and participation in the study. We also thank the faculty and staff of Department of Anatomy of our institute for their co-operation in conduct of this study.

**Conflict of Interest:** None Declared.
